# Type I interferon controls vertical transmission and fetoplacental infection of Oropouche virus

**DOI:** 10.1016/j.isci.2026.114647

**Published:** 2026-01-12

**Authors:** Stefanie Primon Muraro, Gabriela Fabiano de Souza, Yael Alippe, Camila Lopes Simeoni, Aline Vieira, Julia Forato, Paula Mendes Lavagnini, Carolina Manganeli Polonio, Lilian Gomes de Oliveira, Matheus Cavalheiro Martini, Xinyi Hua, Michelle Elam-Noll, William M. de Souza, Luciano Figueiredo Borges, Maria Laura Costa, Jean Pierre Schatzmann Peron, Michael S. Diamond, José Luiz Proenca-Modena

**Affiliations:** 1 Department of Genetics, Microbiology and Immunology, University of Campinas, Campinas, São Paulo, Brazil; 2 Department of Medicine, Washington University School of Medicine, St. Louis, MO, USA; 3 Department of Immunology, University of São Paulo, São Paulo, São Paulo, Brazil; 4 Department of Microbiology, Immunology, and Molecular Genetics, College of Medicine, University of Kentucky, Lexington, KY, USA; 5 Department of Biological Science, Federal University of São Paulo, São Paulo, São Paulo, Brazil; 6 Department of Obstetrics and Gynecology, School of Medical Sciences, University of Campinas, Campinas, São Paulo, Brazil; 7 Department of Molecular Microbiology, Washington University School of Medicine, St. Louis, MO, USA; 8 Department of Pathology & Immunology, Washington University School of Medicine, St. Louis, MO, USA

**Keywords:** Immunology, Virology

## Abstract

The current outbreak of the emerging arthropod-transmitted Oropouche virus (OROV) in South America has been epidemiologically linked to vertical transmissions, microcephaly, and stillbirths. Nevertheless, the impact of OROV infection during pregnancy has not been experimentally evaluated. To address how OROV infection might impact pregnancy outcome, we performed experiments in human cells and mice. Studies in cell cultures showed that the human trophoblast cell lines BeWo and JEG-3 are permissive to OROV infection (strain BeAn19991) and develop type I interferon (IFN)-dependent antiviral response. In our model, loss of type I IFN signaling in the dam resulted in the spread of virus to the placenta and fetus, whereas loss in the fetus alone was not sufficient to cause fetal infection. Collectively, our study shows that placental cells are susceptible to OROV infection and that the outcome for fetus depends on the integrity of the type I IFN immune response in the dam.

## Highlights

• OROV can cross the hematoplacental barrier and cause congenital infection in mice
• Maternal IFNAR1 response limits viral spreading and fetal susceptibility to OROV infection
• Fetal IFNAR1 deletion does not affect fetal susceptibility to OROV infection
• Maternal or fetal IFNLR1 deletion does not affect susceptibility to OROV infection

## Graphical abstract

**Figure:**
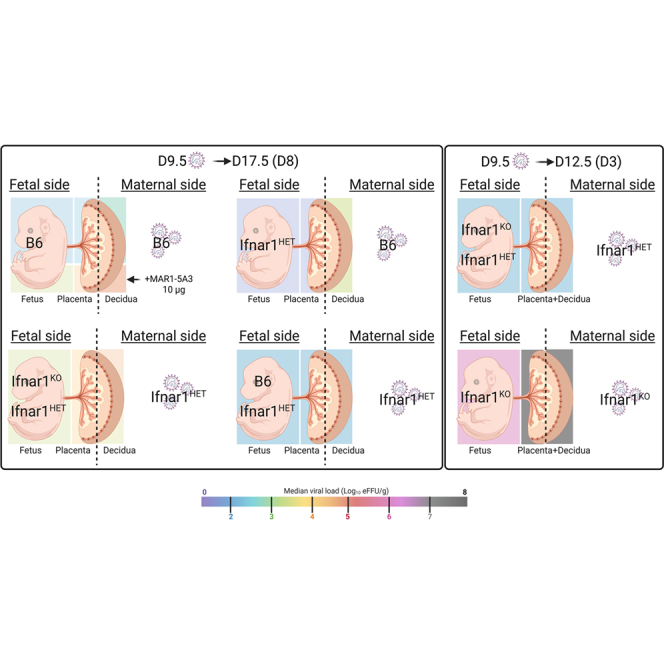

## Introduction

*Orthobunyavirus* *oropoucheense* (Oropouche virus, OROV) is a neglected arbovirus that has been responsible for causing Oropouche fever in Latin America and the Caribbean since its discovery in the 1950s^1^^,^^2^ Over the last year, Oropouche fever re-emerged in the Amazon region in Brazil, Colombia, Peru, and Bolivia, followed by a rapid spread toward the densely populated East Coast of Brazil.^3^^,^^4^^,^^5^ In 2024, OROV spread resulted in autochthonous cases in Cuba and the Dominican Republic, including many cases from travelers returning to Italy, Spain, Germany, and the USA.^6^ Recently, reports of vertical transmission of OROV were published, indicating that the infection in women is linked temporally to miscarriages and fetal microcephaly.^7^^,^^8^^,^^9^

OROV is a tripartite, negative-sense RNA virus in the *Orthobunyavirus* genus of the *Peribunyaviridae* family.^10^ OROV is transmitted mainly by *Culicoides paraensis* midges to pale-throated sloths (*Bradypus tridactylus*), and possibly involves other non-human primates and mammals.^11^^,^^12^ The transmission of OROV in urban settings is due to an epidemic cycle between midges and humans and potentially some mosquito species, including *Culex quinquefasciatus*.^11^^,^^12^^,^^13^^,^^14^^,^^15^^,^^16^

Most OROV infections are mild and self-limited febrile illnesses, with rash, arthralgia, and photophobia.^11^^,^^17^^,^^18^ While acute symptoms usually disappear after a week, some patients develop severe complications that include neurological sequelae (e.g., meningitis, meningoencephalitis, and death), hemorrhagic manifestations, and possibly, pregnancy complications (e.g., stillbirths and congenital anomalies).^9^^,^^11^^,^^17^^,^^18^^,^^19^ To date, relatively few studies have addressed OROV-host interactions and pathogenesis.^20^^,^^21^^,^^22^ Although the responses to type I interferon (IFN) and pattern recognition receptors (e.g., RIG-I-like receptor signaling pathway) are critical to controlling OROV infection,^20^ their effects in pregnancy remain unknown. In this study, we performed experiments in mice and human trophoblast cell lines to investigate the mechanisms of maternal-fetal OROV transmission.

## Results

### OROV infects human placental cell lines

**Figure 1:**
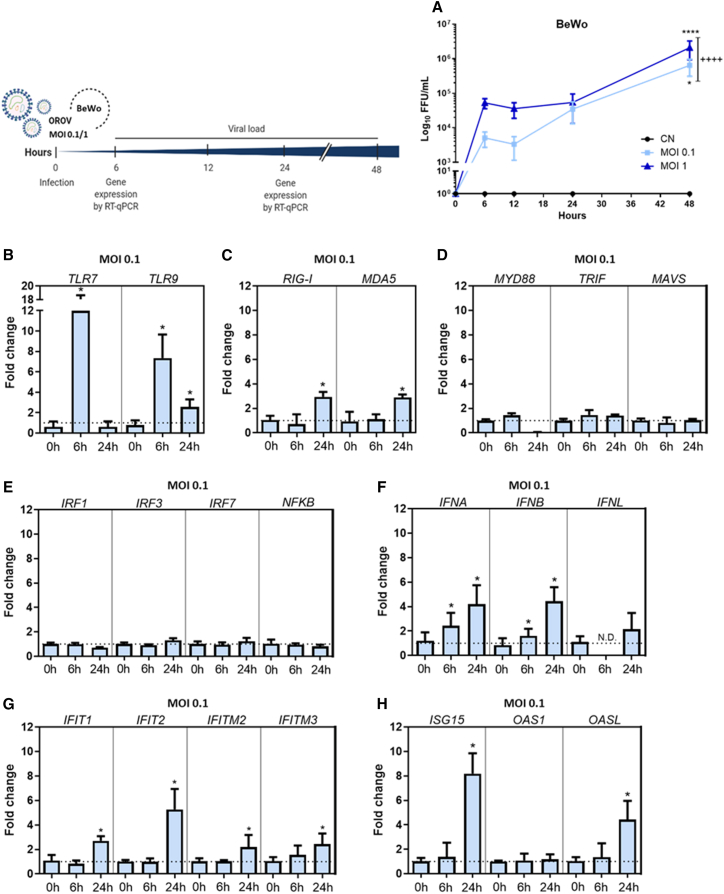
Kinetics of OROV replication in the human placental cell line (BeWo) (A) BeWo cells were inoculated with OROV at two different multiplicities of infection (MOIs = 0.1 and 1). The virus titer in the supernatant was determined by the focus-forming assay at 0, 6, 12, 24, and 48 h after infection. Gene expression at MOI of 0.1 was detected by real-time quantitative PCR, using specific primers and *GAPDH* as a normalizing control. (B–H) Data were analyzed using the comparative CT method (ΔΔCT). Data are expressed as fold increase over the respective control and normalized using the *GAPDH* gene. Data are expressed as the mean ± SD and pooled from two to three independent experiments performed in triplicates. Dashed line corresponds to the detection limit. Data from viral loads were statistically analyzed using two-way ANOVA with Tukey’s posthoc test. For gene expression, mean differences between time points were analyzed using a permutation exact test.∗*p* < 0.05; ∗∗∗∗*p* < 0.001; ^++++^*p* < 0.001 between MOIs of 0.1 and 1. CN, negative control (non-infected cells).

Human cytotrophoblasts and extravillous trophoblasts express low-density lipoprotein receptor-related protein 1 (LRP1), which, recently, has been identified as a receptor for OROV entry and infection.^23^^,^^24^^,^^25^ To determine whether the OROV strain BeAn 19991 can replicate in cells of the human placental origin*,* we characterized OROV replication in BeWo and JEG-3 cells after inoculation at different multiplicities of infection (MOIs = 0.1 and 1) for different times (6-, 12-, 24- and 48-h post-infection [hpi]). Both cells showed peak OROV replication at 48 hpi (Figures 1A and S1A). We next profiled these cells for the expression of host defense genes previously characterized as responsible for OROV control in other tissues.^20^^,^^21^ OROV infection of BeWo cells resulted in increased mRNA expression of Toll-like receptors 7 and 9 (*TLR7* and *TLR9*), *IFNA*, and *IFNB* at 6 hpi and RIG-I-like receptors (*RIG-I* and *MDA5*), *TLR9*, *IFNA*, *IFNB*, IFN-induced proteins with tetratricopeptide repeats (*IFIT1* and *IFIT2*), IFN-induced transmembrane proteins (*IFITM2* and *IFITM3*), and IFN-stimulated genes (*ISG15* and *OASL*) at 24 hpi (Figures 1B–1H). Similarly, JEG-3 cells infected with OROV sustained increased expressions of type I IFNs (*IFNA* and *IFNB*) and type III IFN (*IFNL*) at 6 hpi and IFN-stimulated genes (*ISG15*, *OAS1*, and *OASL*) at 24 hpi (Figures S1D–S1F). These data indicate that the OROV infection induces an antiviral response in these trophoblast-like cells, which leads to higher production of type I and III IFNs and subsequent transcription of antiviral IFN-stimulated genes.

### OROV infects the reproductive organs of female mice

**Figure 2:**
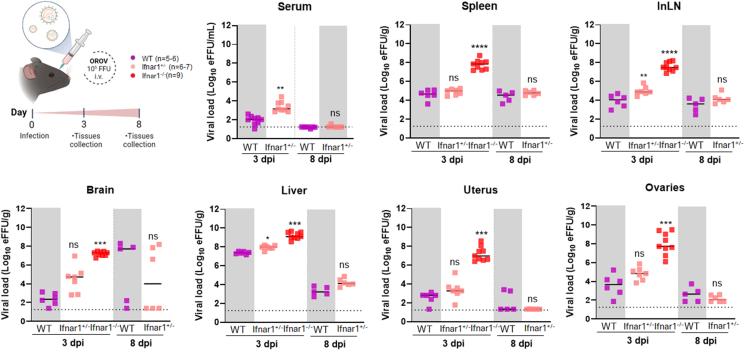
OROV infection in non-pregnant female mice Littermate WT, *Ifnar1*^+/−^, and *Ifnar1*^−/−^ females were challenged via retroorbital route with 10^5^ FFUs of OROV (three independent experiments, *n* = 5–9 mice per group). The indicated tissues were harvested at 3 dpi (WT, *Ifnar1*^+/−^, and *Ifnar1*^−/−^) and 8 dpi (WT and *Ifnar1*^+/−^). Viral loads were assessed by RT-qPCR. Data are presented as median values, and statistical analysis was performed using Kruskal-Wallis, followed by Dunn’s post-test, or one-way ANOVA, followed by Dunnett’s post-test, comparing groups to the WT mice. For pairwise comparisons, Mann-Whitney test was used. ∗∗*p* < 0.01; ∗∗∗*p* < 0.001; ∗∗∗∗*p* < 0.0001; ns, not significant. Dashed line corresponds to the detection limit. eFFUs, equivalent to focus-forming units; dpi, days post infection; i.v, intravenous; inLN, inguinal lymph node.

To investigate whether OROV can infect tissues of the female reproductive tract, littermate non-pregnant *Ifnar1*^+/+^ (wild-type; WT), *Ifnar1*^+/−^, and *Ifnar1*^−/−^ mice were inoculated via retro-orbital injection with 10^5^ focus-forming units (FFUs) of OROV. At 3- or 8-day post-infection (dpi), we measured the viral burden in serum, spleen, inguinal lymph node (InLN), brain, liver, uterus, and ovary after extensive tissue perfusion with PBS. Due to technical reasons, we were unable to recover serum from all *Ifnar1*^−/−^ mice. Viral RNA was detected in serum and multiple tissues of all mouse genotypes (Figure 2). However, *Ifnar1*^−/−^ mice exhibited significantly higher viral loads in the uterus and ovary, as well as in the spleen, InLN, brain, and liver compared with WT mice at 3 dpi. Viral RNA levels were not statistically different between the WT and *Ifnar1*^+/−^ mice at 8 dpi, although the levels were significantly higher in the serum and InLN at 3 dpi, indicating no haploinsufficiency effects (Figure 2). Data are not available for *Ifnar1*^−/−^ mice at 8 dpi because all mice had succumbed to OROV infection by 3 dpi. Early death of the *Ifnar1*^−/−^ mice suggests a critical role for the type I IFN response in controlling OROV mice, as reported previously.^20^

### IFNAR1 controls the maternal-fetal transmission during OROV infection

**Figure 3:**
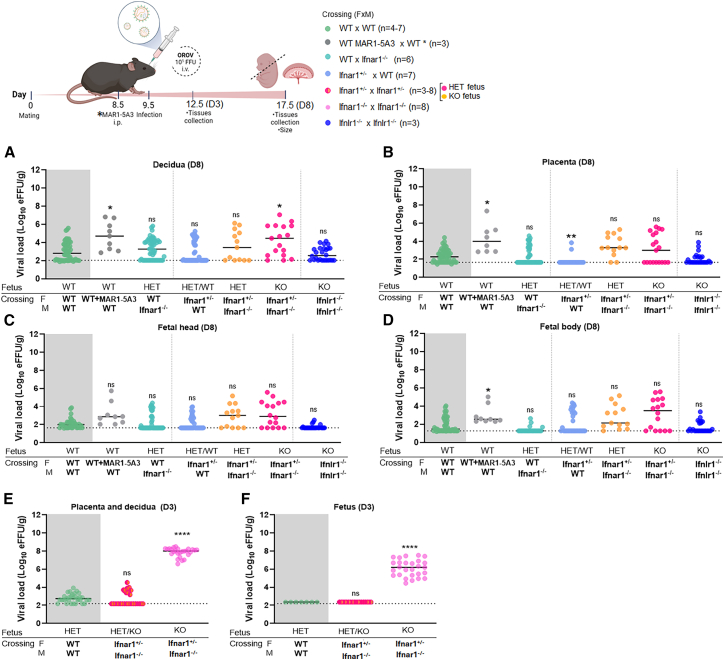
Maternal-fetal interface infection in pregnant mice infected with OROV Pregnant mice after indicated crosses were inoculated via the retroorbital route with 10^5^ FFUs of OROV on day E9.5. Tissues were harvested at 3 dpi on E12.5 or 8 dpi on E17.5. In some animals, 10 μg of MAR1-5A3 was administered via i.p injection one day before infection (E8.5). Viral loads in D8 of the decidua (A), placenta (B), fetal head (C), and fetal body (D) or in D3 of the placenta and its decidua (E) and fetus (F) were assessed by RT-qPCR. Statistical analysis was performed using Kruskal-Wallis, followed by Dunn’s post-test, comparing all groups to the WT mice. ∗*p* < 0.05; ∗∗*p* < 0.01, ∗∗∗∗*p* < 0.0001; ns, not significant. Data are presented as median values and are pooled from at least three independent experiments. Dashed line corresponds to the detection limit.

As type I and III IFNs have protective effects at the maternal-fetal interface against other arboviruses (e.g., Zika virus [ZIKV]),^26^^,^^27^^,^^28^^,^^29^ we investigated the roles of IFNAR1 (Interferon Alpha/Beta Receptor 1) and IFNLR1 (IL28Rα; Interferon Lambda Receptor 1) in controlling the vertical transmission of OROV. For this study, we measured the viral burden in the maternal decidua, fetal placentas and bodies, and tissues of the dam when *Ifnar1*^+/−^ or WT females were mated with *Ifnar1*^−/−^ or WT males or when *Ifnlr1*^−/−^ females were crossed to *Ifnlr1*^−/−^ males. These mating schemes allowed us to assess the role of IFNAR signaling in the fetus and IFNLR signaling in both the dam and fetus showing vertical transmission and infection of OROV. Pregnant mice were inoculated via the retroorbital route with 10^5^ FFUs of OROV on day E9.5 of gestation (after placentation) and euthanized on day E17.5. The maternal decidua from *Ifnar1*^+/−^ × *Ifnar1*^−/−^ crossings showed a small but statistically significant increase in OROV infection (44.8-fold, *p* = 0.02) compared with WT mice. However, there was no significant difference in OROV infection across the fetal placenta, heads, and bodies of *Ifnar*^+/−^ and *Ifnar1*^−/−^ fetuses compared with WT fetuses (Figures 3A–3C). Because of the early death phenotype (day +3) of OROV-infected *Ifnar1*^−/−^ dams, we devised an alternate strategy to inhibit type I IFN signaling in both the dam and fetus, using a blocking anti-IFNAR1 monoclonal antibody (mAb, MAR1-5A3). To determine a relevant dose, we first administered to nonpregnant mice 10–100 μg of MAR1-5A3 intraperitoneally (i.p.). The mice given 30 or 100 μg died at 3 dpi, whereas those given 10 μg survived for 8 days (Figure S2). On the basis of these results, we administered 10 μg of anti-IFNAR1 mAb at day E8.5; this resulted in increased OROV infection in the pregnant mice with slightly higher viral RNA loads in the tissues of the dam, decidua (79.1-fold, *p* = 0.01), placenta (82.5-fold, *p* = 0.01), and fetal body (12.0-fold, *p* = 0.03) (Figures 3A–3C, S3A–S3G). In comparison, no statistically significant differences in viral load were observed between the maternal and fetal tissues from *Ifnlr1*^−/−^ × *Ifnlr1*^−/−^ crosses compared with WT mice, suggesting that type III IFN responses are not sufficient for controlling OROV vertical transmission. Collectively, these data indicated that OROV can replicate in the placenta and cross the placental barrier to reach the fetus. However, in mice, type I IFN-induced response in the dams could restrict OROV replication and limit or prevent spreading to the developing fetus.

To determine whether the relatively low levels of OROV in the fetus at E17.5 (8 dpi) were due to rapid viral clearance, we collected tissues from pregnant mice at 3 dpi (E12.5) and measured viral loads using RT-qPCR (Figures 3E and 3F). At this earlier time point, we were also able to examine tissues from *Ifnar1*^−/−^ × *Ifnar1*^−/−^ crossings, since they were alive even at this time point after OROV infection (Figure S3P). Notably, higher viral loads of decidua and placenta (1.8 × 10^5^-fold; 6.7 × 10^5^-fold, *p* < 0.0001) and fetal tissues (6.7 × 10^3^-fold, *p* < 0.0001) were observed in dams and fetuses from *Ifnar1*^−/−^ × *Ifnar1*^−/−^ crosses at 3 dpi (E12.5) compared with those from WT × WT or *Ifnar1*^+/−^ × *Ifnar1*^−/−^ matings (Figures 3E–3F, S3H–S3M). Collectively, these results indicated that the type I IFN response in the dam controls OROV replication, which impacts dissemination to the placenta and fetal tissues from mice, and that complete deletion of the IFNAR1 gene leads to a higher viral load during OROV infection in pregnant mice.

### OROV infection during pregnancy does not affect fetal morphology

**Figure 4:**
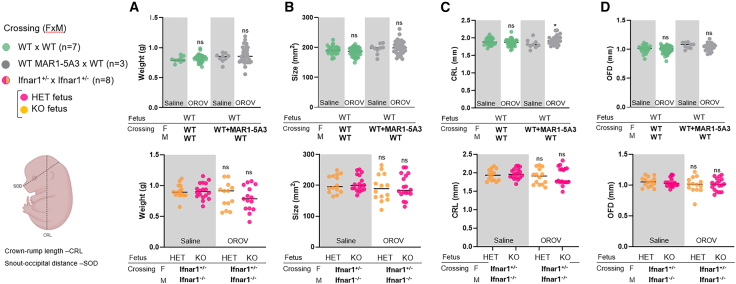
Size and weight of the fetuses after maternal infection with OROV (A) Pregnant mice were inoculated with 10^5^ FFUs of OROV via the retro-orbital route on day E9.5 Eight days later (E17.5), fetuses were harvested for measurements. In some animals, 10 μg of MAR1-5A3 was administered via i.p injection one day before infection (E8.5). Weight of the fetuses was obtained by fetal head and fetal body individual values (A). Size was obtained by measuring the crown-rump length (CRL) and snout-occipital distance (SOD), also individually represented. (B–D) For pairwise statistical comparisons, the Mann-Whitney test was used against its respective mock-infected dam. ∗*p* < 0.05; ns, not significant.. Data are presented as median values and are pooled from at least three independent experiments.

To assess whether maternal-fetal OROV transmission impacts fetal morphology, we performed morphometric analyses by measuring the crown-rump length (CRL) and snout-occipital distance (SOD) from standardized photographs of all fetuses positioned on a dissecting board. Fetal weights from all litters were also recorded to complement these size measurements (Figure 4). Fetuses from *Ifnar1*^+/−^ × *Ifnar1*^−/−^ crossings were slightly smaller (i.e., low weight and small size) than those from the mock-infected group, but this did not attain statistical significance (Figures 4A and 4B). There was no significant reduction, although some differences were observed in CRL measurement of fetuses from the infected WT MAR1-5A3 × WT mice compared with its saline group (Figure 4C). Also, no differences were observed between sex and genotype proportions of the fetuses with or without OROV infection (Table S3). Administration of 10 μg of anti-IFNAR1 mAb to the dam also did not affect fetal weight and size. Fetal viability and fetal reabsorption were assessed in all groups collected at D8, with no significant differences observed between the groups, although there was a trend in *Ifnar1*^+/−^ × *Ifnar1*^−/−^ crossings (Figures S3N–S3O). The histopathological analysis of *Ifnar1*^+/−^ × *Ifnar1*^−/−^ crossing placentas from the control group revealed normal placental architecture, with no detectable alterations in its three main regions (basal plate, chorionic plate, and placental villi) (Figure S4A). In contrast, the examination of infected placentas from *Ifnar1*^+/−^ × *Ifnar1*^−/−^ crossings showed multiple areas of calcification within the placental villi region, along with the presence of trophoblastic cells exhibiting cytopathic effects (Figures S4B–S4E). These data indicated that OROV-induced fetal damage is minimal when the type I IFN response is intact in the dam, regardless of the fetal *Ifnar1* genotype despite evidence of placental damage.

## Discussion

In this study, we demonstrated experimentally that OROV can be transmitted vertically from the mother to the fetus in mice when innate immune responses in the dam are perturbed. Our findings using an anti-IFNAR1 blocking mAb or IFNAR1 deficiencies in the fetus showed that fetal infection does not necessarily lead to major fetal malformations, in contrast to other congenital infections by arboviruses like ZIKV, which can cause anatomic and developmental abnormalities in fetuses.^30^ We also demonstrated that OROV can replicate in human trophoblast cells *in vitro,* infect female reproductive organs in mice, and cross the placental barrier in mice, regardless of the genetic background of the dam, sire, or fetal tissues. We hypothesize that OROV can establish a productive infection in placental cell lines and reproductive tissues due to enriched expression of LRP1, a host factor that enables OROV entry.^23^^,^^24^^,^^25^ Our experiments revealed histopathological alterations in the placentas collected at 8 dpi after OROV, similar to placental dysfunction reported in ZIKV-infected *Ifnar1*^+/−^ mice and Rift Valley fever virus-infected Sprague-Dawley rats.^31^^,^^32^ One major caveat to our experiments is that we were unable to assess the effects on fetal morphology in *Ifnar1*^−/−^ dams at 8 dpi due to early and uniform mortality. However, we observed high infection in fetal bodies at 3 dpi (E12.5).

An intact type I IFN signaling response in the dam was essential for controlling OROV infection in both maternal and fetal tissues, as a deficiency of the type I IFN response in fetal tissues alone did not affect OROV infection. The expression of type I and type III IFN receptors in trophoblasts serves as a key mechanism for restricting ZIKV congenital infection.^29^^,^^33^ While a type I IFN-dependent response in the mouse placenta during ZIKV infection has a deleterious effect on development, leading to an abnormal maternal-fetal barrier architecture,^31^^,^^34^ OROV infection does not appear to elicit such a response. Type III IFN, produced by human trophoblasts, plays an essential role in maintaining the placental barrier during ZIKV infection, acting in both an autocrine and paracrine manner.^35^ Indeed, the human JEG-3 showed increased production of type III IFN after infection with OROV. While we did not observe *in vivo* any significant changes in viral load of *Ifnlr1*^−/−^ mice, it is possible that the protective effects of type III IFNs may be seen in the setting of an absence of type I IFN signaling.

### Limitations of the study

Our study has several limitations. First, there is limited information about the vertical transmission and congenital infection caused by OROV in humans,^7^^,^^9^^,^^36^^,^^37^ which restricts our ability to design similar experiments in mice. For example, further studies should determine the gestational period with greatest risk for maternal-fetal transmission, as previously determined for ZIKV, WNV, murine cytomegalovirus (MCMV), influenza A virus (IAV), and SARS-CoV-2.^27^^,^^31^^,^^38^^,^^39^^,^^40^^,^^41^^,^^42^ A recent study demonstrated that OROV infection induced at mid-gestation is able to cause brain vacuolation in several fetuses in mice.^43^ Second, we did not investigate earlier gestational timepoints in mice before placentation was complete. All experiments were performed at E9.5, which may limit our conclusions regarding the gestational impact of OROV, since human placental explants collected during early pregnancy have been shown to sustain higher viral titers than explants obtained from placentas at later gestational stages.^44^ Third, due to differences in placental development and the composition of the blood-placental barrier between mice and humans, the phenotypes observed in mice may not directly compare to humans. Fourth, our *in vitro* experiments were conducted exclusively using the established human placental cell lines, which likewise do not recapitulate the full complexity of the human placenta, although recent data indicate that OROV can replicate in 3D trophoblast organoids and human villous explants.^44^ Fifth, OROV infection in WT immunocompetent mice does not recapitulate the disease observed in humans, likely due to a species-specific failure to antagonize mouse innate immune responses.^20^^,^^21^^,^^45^ Sixth, OROV isolates from the 2023–2024 outbreak showed increased virulence in Vero CCL81, Huh7, and U-251 cell lines, though their virulence was not tested in trophoblastic cells lines.^46^ The new OROV isolate resulted from reassortment between two related orthobunyaviruses and comprised the M segment from Brazilian isolates (2009–2018) and L and S segments from the isolates circulating in Peru, Colombia, and Ecuador during 2008–2021.^4^^,^^46^ The contemporary OROV strains exhibit enhanced growth, polymerase activity, virulence, and immune escape.^43^ Although the vertical transmission of OROV has been reported recently, the historical OROV strain has already showed placental tropism.^44^ However, the production of neutralizing antibodies during maternal infection with ancestral OROV lineages confer only partial protection to offspring after infection with contemporary OROV strains during gestation in mice.^43^ Thus, pregnancy infection studies in other animal models, such as Syrian golden hamsters, could provide insights into the consequences of vertical transmission of OROV.

In summary, our data highlight the potential vertical transmission and congenital infection of OROV. We demonstrated that the type I IFN response in maternal tissues is essential to control OROV infection, and an absence of type I IFN signaling pathway enables the generation of a viral burden that spreads readily across the maternal-fetal interface. Further studies are needed to determine additional factors that modulate the linkage between OROV infection and possible adverse pregnancy outcomes.

## Resource availability

### Lead contact

Further information and requests for resources and reagents should be directed to and will be fulfilled by the lead contact, José Luiz Proenca-Modena (jlmodena@unicamp.br).

### Materials availability

Requests for materials and resources from the study should be directed to the lead contact.

### Data and code availability

• All data reported in this paper will be shared by the lead contact upon request and is also available at Repositório de Dados de Pesquisa da Unicamp (REDU) https://doi.org/10.25824/redu/WLYNVT. No new viral sequences were generated or deposited in this study. The OROV strain BeAn 19991 used in this study is identical to the one deposited in GenBank under accession numbers GenBank: KP_052850, KP_052851, and KP_052852 for the L, M, and S segments, respectively (key resources table).
• This paper does not report original code. No custom code or algorithms were developed for analysis software such as the R project.
• Any additional information required to reanalyze the data reported in this paper is available from the lead contact upon request.

## Acknowledgments

This work was carried out with support of the 10.13039/100015748São Paulo Research Foundation (grant no. 2016/00194-8). J.L.P.-M. was supported by the 10.13039/501100005993National Council for Scientific and Technological Development (CNPq, grant no. 309971/2023-3). S.P.M. was supported by 10.13039/501100001807FAPESP (grant nos. 2018/13645-3 and 2021/10615-9), 10.13039/501100002322Coordenação de Aperfeiçoamento de Pessoal de Nível Superior - Brazil (Financing Code 001), CNPq (162104/2018-9), and 10.13039/501100006417Fundo de apoio ao ensino, 10.13039/501100006417pesquisa e extensão da Unicamp (FAEPEX, 21814-24). WMdS was supported by 10.13039/100010269Wellcome Trust – Digital Technology Development Award in Climate Sensitive Infectious Disease Modeling (grant no. 226075/Z/22/Z). We thank the staff of the 10.13039/100002147Life Sciences Core Facility (LaCTAD) from 10.13039/100004871State University of Campinas (UNICAMP) for the cell biology analysis. Thanks to Eurico Arruda (University of São Paulo, Ribeirão Preto, Brazil) for providing the OROV strain BeAn 19991.

## Author contributions

Conceptualization, S.P.M., M.D., and J.L.P.-M.; methodology, S.P.M., G.F.d.S., Y.A., C.L.S., A.V., J.F., P.M.L., C.M.P., L.G.d.O., M.C.M., and M.E.N.; investigation, S.P.M., G.F.d.S., Y.A., J.P.S.P., M.L.C., L.F.B., M.D., and J.L.P.-M.; writing – original draft, S.P.M.; writing – review & editing, W.M.d.S., M.D., and J.L.P.-M.; funding acquisition, M.D. and J.L.P.-M.; resources, M.D. and J.L.P.-M.; supervision, M.D. and J.L.P.-M.

## Declaration of interests

M.S.D. is a consultant or advisor for Inbios, Vir Biotechnology, IntegerBio, Moderna, Merck, and GlaxoSmithKline. The Diamond laboratory has received unrelated funding support in sponsored research agreements from Vir Biotechnology, Emergent BioSolutions, and IntegerBio.

## STAR★Methods

### Key resources table

**Table:** 

REAGENT or RESOURCE	SOURCE	IDENTIFIER
**Antibodies**		

IFNAR-1 Monoclonal Antibody (MAR1-5A3)	Leinco Technologies	Cat#I-401; RRID: AB_2830666
Oropouche virus immune ascitic fluid	ATCC	Cat#VR-1228AF
Goat Anti-Mouse IgG H&L (HRP)	Abcam	Cat#ab6789; RRID: AB_955439

**Bacterial and virus strains**		

OROV strain BeAn 19991	Provided by Prof. Dr. Luiz Tadeu Morais Figueiredo from the Medical School of the University of São Paulo, Ribeirão Preto, Brazil.	GenBank: KP052850, KP052851 and KP052852

**Chemicals, peptides, and recombinant proteins**		

Dulbecco’s Modified Eagle Medium (DMEM)	Gibco	Cat#11995-040
RPMI 1640 Medium	Gibco	Cat#11875-093
Fetal Bovine Serum	Omega Scientific	Cat#FB-01
Dulbecco`s Phosphate Buffered Saline (PBS)	Sigma-Aldrich	Cat#D8537
Methyl cellulose	Sigma-Aldrich	Cat#M0512
Paraformaldehyde 20% Solution (PFA)	EMS	Cat#15713-S
TrueBlue detection reagent	KPL	Cat#5510-0030
Hematoxylin	Vector	Cat#H-3401
Eosin	Sigma-Aldrich	Cat#HT110132
Penicillin-streptomycin	Gibco	Cat#15140-122

**Critical commercial assays**		

5× MagMax Viral Isolation kit	Applied Biosystems	Cat#AMB18365
TaqMan RNA-to-CT 1-Step kit	Thermo Fisher	Cat#4392938
OROV PrimeTime qPCR Assay	IDT	Cat#427491414
High-Capacity cDNA Reversion Transcription kit	Applied Biosystems	Cat#4374966
iTaq Universal SYBR Green Supermix	BioRad	Cat#1725121
Q5′-Hot Start High-Fidelity 2x Master Mix kit	New England BioLabs	Cat#LM0494L

**Experimental models: Cell lines**		

BeWo (human choriocarcinoma cells)	ATCC	Cat#CCL-98
JEG-3 (human choriocarcinoma cells)	ATCC	Cat#HTB-3
Vero E6	ATCC	Cat#CRL-1586

**Experimental models: Organisms/strains**		

C57BL/6	Jackson Laboratory	Cat#000664
Ifnlr1-/-	Jackson Laboratory	See Ank et al.^47^
Ifnar1-/-	Jackson Laboratory	See Muller et al.^48^

**Oligonucleotides**		

Table S1. Primers for gene expression	N/A	See Table S1
Table S2. Primers for genotyping	N/A	See Table S2

**Software and algorithms**		

Biorender	Biorender	N/A
GraphPad Prism version 10	GraphPad Software	N/A
ImageJ software	National Institutes of Health	N/A

**Other**		

BD Microtainer	BD	Cat#365967
Zirconia/Silica Beads	Biospec	Cat#11079110z

### Experimental model and study participant details

#### Animals and ethical approval

In all experiments, female mice of 6-8 months old were used. Wild-type (WT) C57BL/6 mice lineage was purchased from Jackson Laboratories, USA. Congenic *Ifnar1*^-/-^ and *Ifnlr1*^-/-^ mice were used as previously described.^47^^,^^48^ All mice were bred in a specific-pathogen-free facility at the University of Campinas or Washington University School of Medicine in St. Louis. OROV infections were performed on embryonic day E9.5. Intravenous inoculations in pregnant dams were performed by retro-orbital injection with 10^5^ focus-forming units (FFU) of OROV in a volume of 100 μl. When indicated, 10 μg of anti-IFNAR1 monoclonal antibody MAR1-5A3 was administered via intraperitoneal injection one day before infection. OROV infection experiments were performed with 5- to 6-week-old female mice. This study was approved and followed the recommendations in the Guide for the Care and Use of Laboratory Animals of the National Institutes of Health after approval by the Institutional Animal Care and Use Committee at the Washington University School of Medicine in St. Louis, USA (protocol 21-0246) and Comitê de Ética no Uso de Animais of University of Campinas (protocol 5302-1/2019).

#### Cells

Vero E6, BeWo (human choriocarcinoma cells; ATCC CCL-98) and JEG-3 (human choriocarcinoma cells; ATCC HTB-36) used in the study were purchased from ATCC (USA). Vero E6 cells (ATCC CRL-1586) were cultured in Dulbecco’s modified Eagle’s medium (DMEM), BeWo and JEG-3 in RPMI 1640 medium supplemented with 10% fetal bovine serum (FBS) and 1% Penicillin-streptomycin (10000 IU/mL penicillin and 200 mM streptomycin) at 37°C with 5% CO_2._ Cell lines were tested for mycoplasma contamination by PCR.

### Method details

#### Cell infection experiments

Experiments using BeWo and JEG-3 cells were performed in 24-well plates using 2x10^5^ cells/mL. Cells were infected with OROV using a MOI of 0.1 and 1 for 2 h in DMEM medium without FBS. Next, the cells were washed with phosphate-buffered saline (PBS) and supplemented with DMEM with 2% FBS. Subsequently, cells were incubated for up to 48 h.

#### Virus and biosafety setting

OROV strain BeAn 19991 (GenBank with accession numbers KP052850, KP052851 and KP052852 for the L, M and S segments, respectively) was produced in Vero E6 cells. OROV experiments were conducted under enhanced biosafety level 2 (BSL2) and animal BSL2 (A-BSL2) containment at the University of Campinas (Brazil) or Washington University School of Medicine in St. Louis (USA).

#### Viral inoculation and tissue collection

Scheduled pregnancies were established, and embryonic day E0.5 (i.e., 0.5 days post-conception) was determined after the detection of vaginal plugs. Females were inoculated on day E9.5 with 10^5^ FFU (WT, *Ifnar1*^+/-^ and *Ifnlr1*^-/-^), 10^2^ FFU (*Ifnar1*^-/-^) or cell culture supernatant (mock-infected) intravenously by retro-orbital route under isoflurane anesthesia. Mice were euthanized on days E12.5 (3 dpi) or E17.5 (8 dpi). Maternal and fetal tissues were collected on a cooled surface and immediately stored on dry ice. Animals were not perfused due to the technical difficulty of performing perfusion in pregnant females. The decidua was dissected from the fetal placenta as previously described.^49^ Serum collection was performed by cardiac puncture in a blood collection tube (Becton Dickinson, USA), centrifuged in 2000 × g for 6 minutes at 4°C, and aliquoted into a new tube. To determine fetal size, images of the fetuses obtained at the time of collection were analyzed using the ImageJ software, considering the crown-rump length (CRL) and the snout-occipital distance (SOD) using a dissecting board. All samples were stored at -80°C.

#### RNA extraction and RT-qPCR

The tissues were weighed and homogenized in tubes containing zirconia spheres and DMEM medium using MagNA Lyser Instrument (Roche LifeScience, USA). Homogenates were centrifuged at 10,000 × g for 10 min, and supernatant was collected for RNA extraction using the 5× MagMax Viral Isolation kit on a Kingfisher Flex extraction robot (Thermo Scientific, USA) following the manufacturer instructions.

For viral loads, RNA was amplified using the TaqMan RNA-to-CT 1-Step kit and PrimeTime qPCR Assay specific for the OROV S segment.^50^ Samples were amplified on a QuantStudio 6 Flex System (Applied Biosystems, USA) as follows: 48°C for 15 min followed by 2 min at 95°C; then 40 cycles of 95°C for 15 sec and 60°C for 1 min. The amount of RNA was expressed as viral RNA equivalents per gram (tissue) or mL (serum) after interpolation on a standard curve of serial dilutions of OROV RNA obtained from a stock with known concentration in FFU/mL.

For gene expression (Table S1), RNA samples were reverse transcribed with High-Capacity cDNA Reversion Transcription kit using SimpliAmp thermocycler (Thermo Fisher Scientific) according to the manufacturer’s instructions. After cDNA quantification, samples were normalized to 500 ng/uL of cDNA. RT-qPCR was performed using iTaq Universal SYBR Green Supermix under the following conditions: 95°C for 3 min and 35 cycles of denaturation at 95°C for 15 sec and annealing-extension at 60°C for 1 min. The glyceraldehyde 3-phosphate dehydrogenase (GAPDH) gene was used as an endogenous control. Quantitation was determined relative to uninfected cells, after normalization with the chosen endogenous control using the 2−ΔΔCt method.

#### Genotyping

Genotyping of adult mice and fetuses were performed in approximately 1 mm^2^ of mouse tail digested in alkaline lysis buffer (25 mM NaOH and 0.2 mM EDTA, pH 12) for 15 min at 95°C and neutralization buffer (40 mM Tris-HCl pH 5). Then, samples were centrifuged at 10,000 x g for 10 min and genotyped and sexed by conventional PCR (Table S2). PCR was performed using the Q5′-Hot Start High-Fidelity 2x Master Mix kit following the manufacturer’s instructions. The PCR product obtained were loaded in a 2% agarose gel. The gel was visualized on the iBright imaging system (Thermo Fisher Scientific).

#### Virus titration

For OROV titration by focus forming assay (FFA), samples were thawed, centrifuged by 2,000 × g at 4°C for 10 min, and diluted serially prior to infection in Vero cells. Followed by a 2-h infection period, the cells were washed with PBS and overlayed with semi-solid media containing 1% methylcellulose, DMEM, 4% FBS and 1% Penicillin/Streptomycin. After 24 h of infection, cells were fixed overnight with 4% paraformaldehyde in PBS. Infected cell foci were detected after incubation with a 1:1,000 dilution of polyclonal mouse anti-OROV (Oropouche virus immune ascitic fluid) for 2 h at room temperature. After three washes with permeabilization-wash buffer (P-W; PBS, 0.1% saponin, and 0.1% bovine serum albumin [BSA]), the samples were incubated with a 1:2,000 dilution of horseradish peroxidase (HRP)-conjugated goat anti-mouse IgG for 1 h at room temperature. After three additional washes with P-W buffer, staining was visualized by adding the TrueBlue detection reagent, and the spots were analyzed with a Biospot counter (Cellular Technology) using Immunocapture software. All results were converted into FFU per gram or mL.

#### Histology

Placentas from each group were harvested on E17.5, fixed in 4% PFA in PBS for 24 h and stored at 4°C in ethanol 70% until embedded in paraffin. Samples were submitted to the Histology Core at Washington University School of Medicine in St. Louis for sectioning. For histology, sections obtained from paraffin samples were stained with hematoxylin and eosin. Placental sections were digitized on a Hamamatsu NanoZoomer (HT) at 20X resolution.

### Quantification and statistical analysis

#### Software and data analysis

Data were analyzed using GraphPad Prism version 10 (GraphPad Software, USA). Images were analyzed using ImageJ software (NIH, USA). For virus titration, spots were counted using Immunocapture software (Cellular Technology Limited, USA). Statistical analyses are described in the legends using p values ≤ 0.05 to indicate statistically significant differences between groups and proportionally represented by symbols. Shapiro-Wilk test was used to assess population normality. For group comparisons, we used Kruskal-Wallis followed by Dunn’s post-test or one-way ANOVA followed by Dunnett`s post-test. For pairwise comparisons we used a Mann-Whitney test. Survival curves were compared using the log-rank (Mantel–Cox) test. To compare mean differences in relative gene expression across time points, we applied an exact permutation test, which assesses variations in both distribution and magnitude of the observed values without assuming data normality and is appropriate for analyses involving small sample sizes. Graphical illustrations were made using BioRender (BioRender.com). The number of animals are described in each figure along with the groups.
