# Supplementary material for: Type I interferon controls vertical transmission and fetoplacental infection of Oropouche virus
Source: iScience. 2026 Jan 12;29(2):114647. doi: 10.1016/j.isci.2026.114647 (PMC12918160; doi:10.1016/j.isci.2026.114647)
Supplement: Document S1. Figures S1–S4 and Tables S1–S3 [file mmc1.pdf]

iScience, Volume *29*

Supplemental information

Type I interferon controls vertical transmission and fetoplacental infection of Oropouche virus

Stefanie Primon Muraro, Gabriela Fabiano de Souza, Yael Alippe, Camila Lopes Simeoni, Aline Vieira, Julia Forato, Paula Mendes Lavagnini, Carolina Manganeli Polonio, Lilian Gomes de Oliveira, Matheus Cavalheiro Martini, Xinyi Hua, Michelle Elam-Noll, William M. de Souza, Luciano Figueiredo Borges, Maria Laura Costa, Jean Pierre Schatzmann Peron, Michael S. Diamond, and José Luiz Proenca-Modena

SUPPLEMENTAL INFORMATION

Document S1. Figures S1–S4. Tables S1-S3.

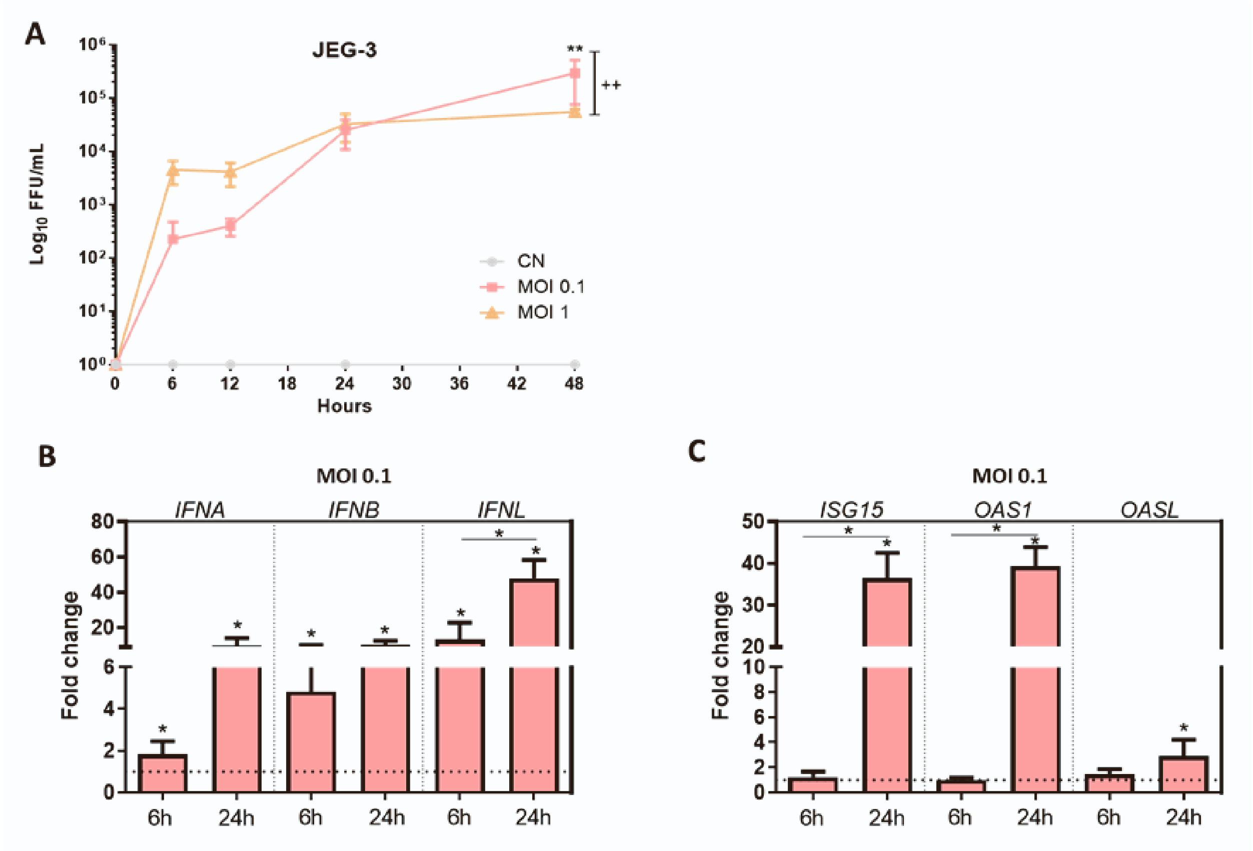

Figure S1. Kinetics of OROV replication in JEG-3 cells. JEG-3 cells were inoculated with two different multiplicities of infection (MOI = 0.1 or 1) of OROV. The virus titer in the supernatant was determined by RT-qPCR and focus-forming assay at 0, 6, 12, 24 and 48 h after infection (**A**). Gene expression at MOI of 0.1 was detected by real-time quantitative PCR using specific primers and *GAPDH* as an endogenous control (**B and C**). Data were analyzed using the comparative CT method (ΔΔCT). Data are expressed as fold increase over the respective control and normalized using *GAPDH*. Data are pooled from 2 independent experiments performed in triplicate and represent the mean values ± SD. Dashed line corresponds to the detection limit. Data from viral loads were statistically analyzed using two-way ANOVA with Tukey's post-hoc test. For gene expression, mean differences between time points were analyzed using permutation exact **test. *p<0.05**; **p<0.01; $^{++}$p<0.01 between MOI 0.1 and 1.

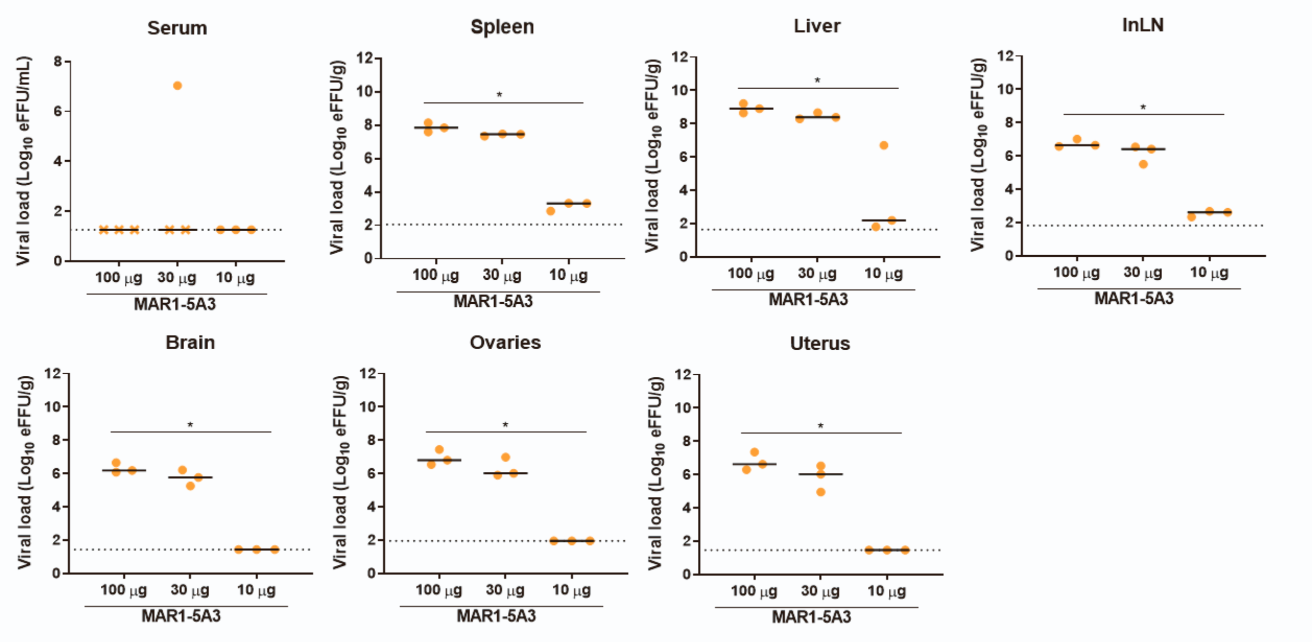

Figure S2. Dose-dependent type I IFN signaling blocking during OROV infection. MAR1-5A3 was administered via i.p injection one day before infection at 100 µg, 30 µg or 10 µg. Mice were inoculated with 10^3 FFU of OROV via retroorbital injection and followed up to 8 days. Viral loads in tissues were assessed at 3dpi (100 µg and 30 µg) or at 8dpi (10 µg). by RT-qPCR. Data are presented as median values and statistical analysis was performed using Kruskal-Wallis followed by Dunn's post-test comparing all groups to WT mice. *$p < 0.05$. Data are representative of one experiment. Dashed lines correspond to limit of detection. X symbol represents dead mice.

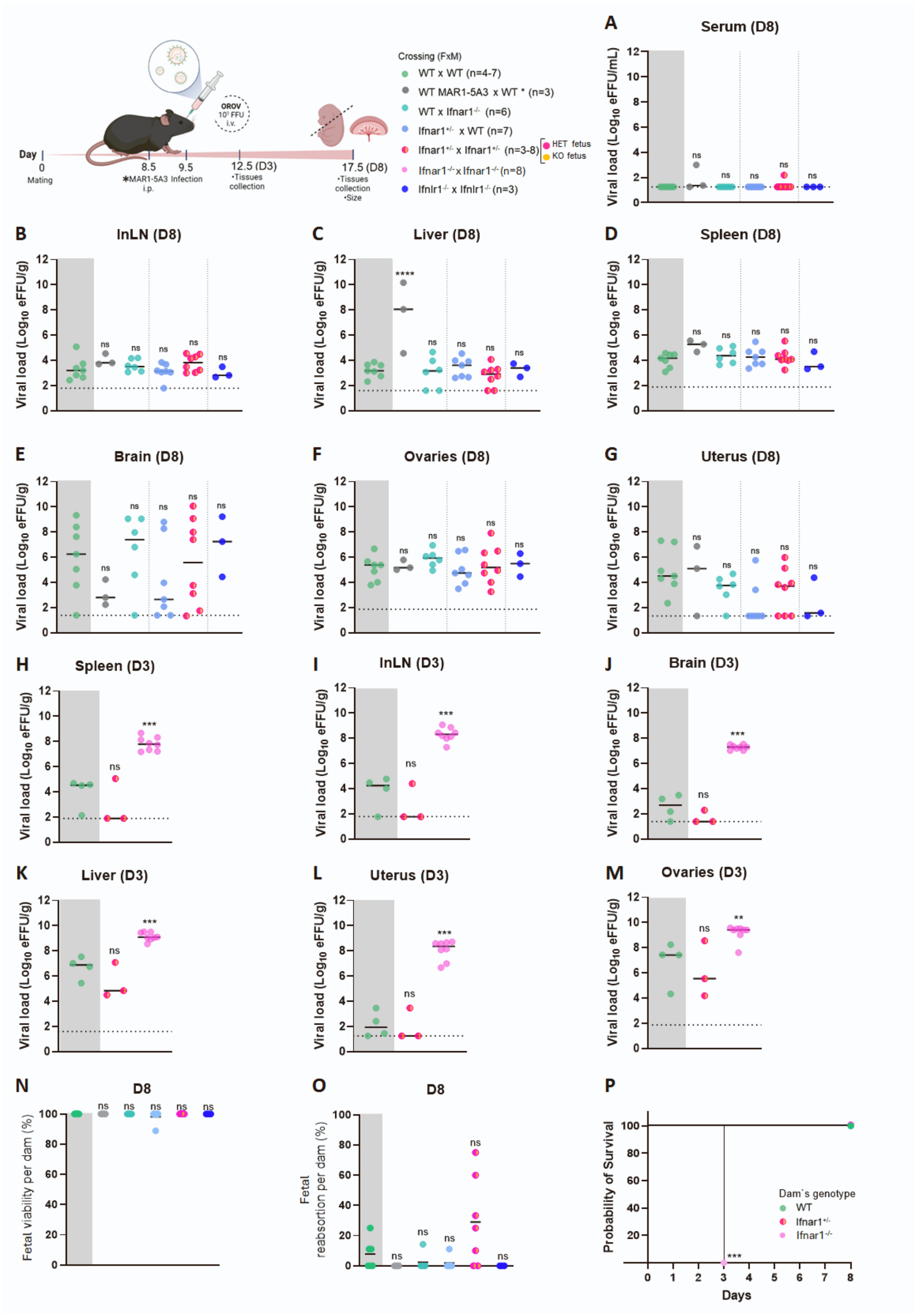

Crossing (FxM)
WT x WT (n=4-7)
WT MAR1-5A3 x WT * (n=3)
WT x Ifnar1-/- (n=6)
Ifnar1+/- x WT (n=7)
Ifnar1+/- x Ifnar1+/- (n=3-8)
HET fetus
KO fetus
Ifnar1-/- x Ifnar1-/- (n=8)
Ifnlr1-/- x Ifnlr1-/- (n=3)
OROV 10^5 FFU i.v.
Day
0 Mating
8.5 *MAR1-5A3 i.p.
9.5 Infection
12.5 (D3) •Tissues collection
17.5 (D8) •Tissues collection •Size
A
Serum (D8)
Viral load (Log10 eFFU/mL)
B
InLN (D8)
C
Liver (D8)
D
Spleen (D8)
E
Brain (D8)
F
Ovaries (D8)
G
Uterus (D8)
H
Spleen (D3)
I
InLN (D3)
J
Brain (D3)
K
Liver (D3)
L
Uterus (D3)
M
Ovaries (D3)
Viral load (Log10 eFFU/g)
ns
****
***
**
N
D8
Fetal viability per dam (%)
O
D8
Fetal reabsortion per dam (%)
P
Probability of Survival
Days
Dam`s genotype
WT
Ifnar1+/-
Ifnar1-/-

Figure S3. OROV infection in maternal tissues. Pregnant mice were inoculated with 10^5 FFU of OROV via retroorbital injection on E9.5. When indicated 10 µg of MAR1-5A3 was administered via i.p injection one day before infection. Viral loads in tissues of the dam at 8 dpi (A-G) or 3 dpi (H-M) were assessed by RT-qPCR. Fetal viability and reabsorption were assessed at D8 (N-O). Dam`s survival was observed up to D8 (experiment endpoint) (P). Data are presented as median values and statistical analysis was performed using Kruskal-Wallis followed by Dunn's post-test or one-way ANOVA followed by Dunnett`s post-test comparing all groups to WT mice. Survival curves were compared using the log-rank (Mantel–Cox) test. *p <0.05; ***p<0.001. Data are representative of at least independent experiments. Dashed lines correspond to limit of detection.

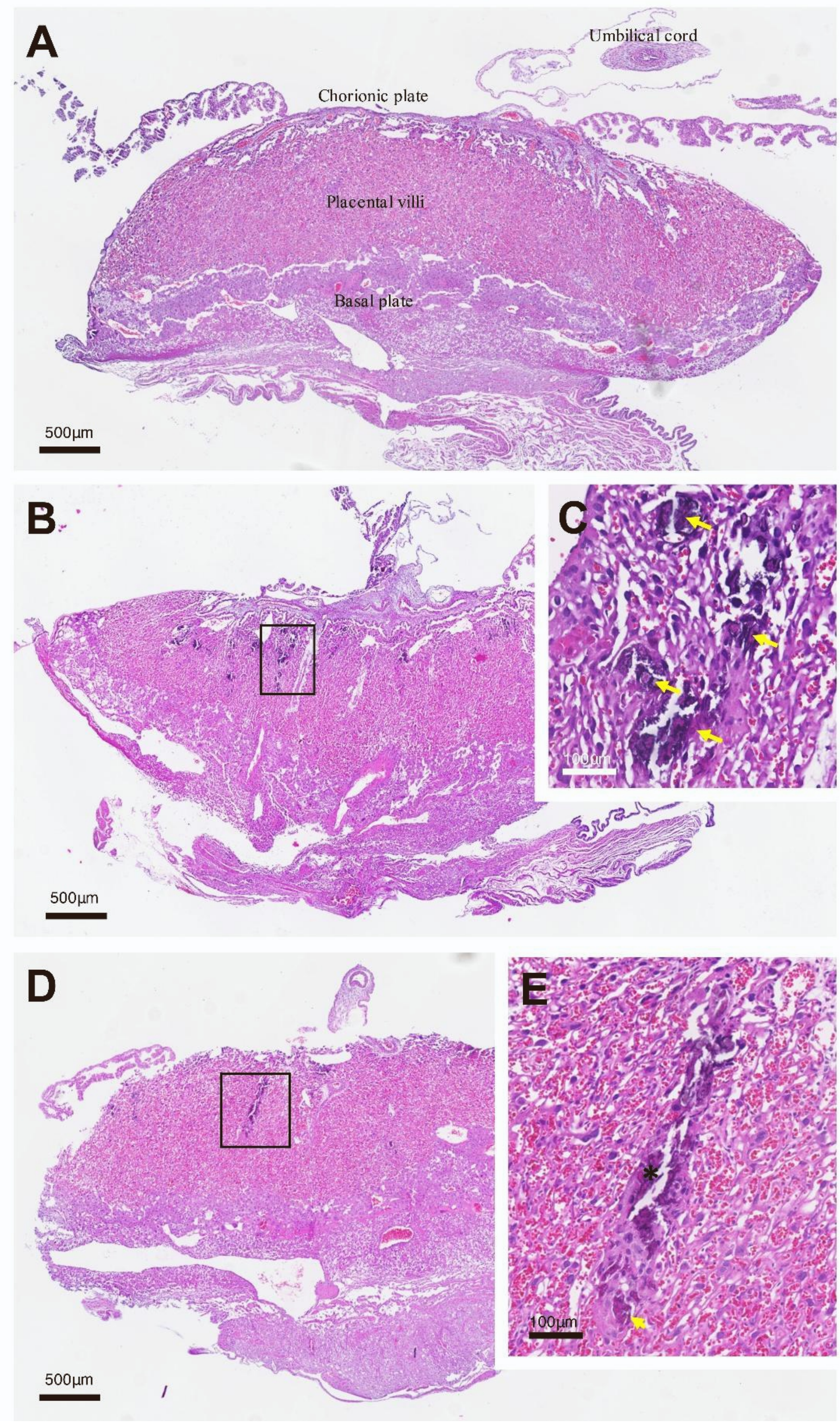

Figure S4. Representative longitudinal section of E17.5 placentas after OROV infection of *Ifnar1*$^{+/-}$ x *Ifnar1*$^{-/-}$ matings. Pregnant mice were inoculated with 10^5 FFU of OROV via retroorbital injection on E9.5.

At E17.5, placentas were harvested for histology. Hematoxylin and eosin staining of placentas was performed on paraffinized samples. The placenta from the control group (**A**) shows normal histological architecture. In contrast, the placenta from the infected group (**B**) presents a region with an area of calcification (rectangle). A higher magnification of this region (**C**) reveals distinct areas of calcification (arrows). Another placenta from the experimental group (**D**) also shows a region with calcification (rectangle), and at higher magnification (**E**), a large, calcified area (asterisk) can be observed, along with the presence of a putative cell exhibiting cytopathic effect (short arrow). Scale bars, 500 μm and 100 μm (higher magnification). Representative images of 3 saline and 4 OROV-infected placentas.

Table S1. Primers and probes used for quantification of gene expression

Target	Forward	Reverse	Ref
Gapdh	CCCATGTTCGTCATGGGTGT	TGGTCATGAGTCCTTCCACGATA	1
Ifit1	AGAAGCAGGCAATCACAGAAAA	CTGAAACCGACCATAGTGGAAAT	2
Ifit2	GGTCTCTTCAGCATTTATTGGTG	TGCCGTAGGCTGCTCTCCA	3
Ifitm2	ATCCCGGTAACCCGATCAC	CTTCCTGTCCCTAGACTTCAC	4
Ifitm3	ATGTCGTCTGGTCCCTGTTC	GTCATGAGGATGCCCAGAAT	5
Ifn-α	TCCATGAGVTGATBCAGCAGA	ATTTCTGCTCTGACAACCTCCC	6
Ifn-β	GCTTGGATTCCTACAAAGAAGCA	ATAGATGGTCAATGCGGCGTC	7
Ifn-λ 2/3	CTGCCACATAGCCCAGTTCA	AGAAGCGACTCTTCTAAGGCATCTT	8
Irf1	CGAATCGCTCCTGCAGCAGA	GCCCAGCTCCGGAACAAACA	9
Irf3	AGCAGAGGACCGGAGCAA	AGAGGTGTCTGGCTGGGAAA	10
Irf7	TGTGCCGAGTGCACCTAGAG	GAAGCACTCGATGTCGTCATAGAG	10
Isg15	TGGTGAGGAATAACAAGGGC	CAGATTCATGAACACGGTGC	2
Mavs	GTCACTTCCTGCTGAGA	TGCTCTGAATTCTCTCCT	11
Mda5	CCAAAGCTGAAGAACACAT	ATCTTCTCTGGTTGCATCT	12
Myd88	GACGACGTGCTGCTGGAGCTG	GATGAAGGCATCGAAACGCTCAG	13
NFkb/p65	ATCCCATCTTTGACAATCGTGC	CTGGTCCCGTGAAATACACCTC	14
Oas1	CAAGCTCAAGAGCCTCATCC	TGGGCTGTGTTGAAATGTGT	2
Oasl	GGGACAGAGATGGCACTGAT	AAATGCTCCTGCCTCAGAAA	2
Rig-I	TGTGCTCCTACAGGTTGTGGA	CACTGGGATCTGATTCGCAAAA	15
Tlr3	TGGTTGGGCCACCTAGAAGTA	TCTCCATTCCTGGCCTGTG	16
Tlr7	CTCCCTGGATCTGTACACCTGTGAG	CTCCCACAGAGCCTTTTCCGGAGCT	17
Tlr9	TTATGGACTTCCTGCTGGAGGTGC	CTGCGTTTTGTCGAAGACCA	18
Trif	GGCCCATCACTTCCTAGCG	GAGAGATCCTGGCCTCAGTTT	19

Table S2. Primers and probes used for mouse genotyping

Target	Primer sequence	Size (bp)
Ifnar1	F:UM4 (Common):<br>5`-AAGATGTGCTGTTCCCTTCCTCGCTCTCA-3`	WT: 150<br>KO: 180
	R1:UM5 (WT):<br>3`-ATTATTAAAAGAAAAGACGAGGCGAAGTGG-5`<br>R2:Neo P3 (KO):<br>3`-ATTCGCAGGGCATCGCCTTCTATCGCC-5`	
Sry	F: 5`-CATGAACGCATTCATCGTGTGGTC-3`	280
	R: 3`-CTGCGGGAAGCAAACTGCAATTCT T-5`	

Table S3: Fetal genotype and sex proportions of each crossing.

F x M	Saline-sex		OROV- sex		p value	Saline-genotype			OROV-genotype			p value
	F %±SD	M %±SD	F %±SD	M %±SD		WT %±SD	HET %±SD	KO %±SD	WT %±SD	HET %±SD	KO %±SD	
WT x WT	38.46±1.50 (5/13)	61.54±1.00 (8/13)	51.16±1.49 (22/43)	48.84±1.46 (21/43)	ns	100±0.00 (13/13)	-	-	100±0.00 (43/43)	-	-	ns
WT x Ifnar$^{-/-}$	26.67±0.00 (4/15)	73.33±1.50 (11/15)	50.00±1.60 (26/52)	50.00±1.70 (26/52)	ns	-	100±0.00 (15/15)	-	-	100±0.00 (52/52)	-	ns
Ifnar$^{-/-}$ x WT	75.00±3.4 (9/12)	25.00±0.50 (3/12)	52.73±2.75 (29/55)	38.18±1.06 (21/55)	ns	41.67±2.50 (5/12)	58.33±1.55 (7/12)	-	47.27±2.25 (26/55)	43.64±1.99 (24/55)	-	ns
Ifnar$^{-/-}$ x Ifnar$^{-/-}$	37.50±0.82 (12/32)	62.5±2.05 (20/32)	23.00±1.83 (20/43)	20.00±1.41 (20/43)	ns	-	43.75±0.94 (13/32)	56.25±1.41 (18/32)	-	41.86±1.39 (18/43)	58.14±2.03 (25/43)	ns

*Chi-square test. Samples were compared to mock of each crossing. F-female. M-male. WT-wild-type. HET-heterozygous. KO-knockout

REFERENCES

1.Micke, P., Ohshima, M., Tahmasebpoor, S., Ren, Z.-P., Östman, A., Pontén, F., and Botling, J. (2006). Biobanking of fresh frozen tissue: RNA is stable in nonfixed surgical specimens. Laboratory Investigation 86, 202–211. 10.1038/labinvest.3700372.

2. Santer, D.M., Minty, G.E.S., Mohamed, A., Baldwin, L., Bhat, R., Joyce, M., Egli, A., Tyrrell, D.L.J., and Houghton, M. (2017). A novel method for detection of IFN-lambda 3 binding to cells for quantifying IFN-lambda receptor expression. Journal of Immunological Methods 445, 15–22. 10.1016/j.jim.2017.03.001.

3.Imaizumi, T., Numata, A., Yano, C., Yoshida, H., Meng, P., Hayakari, R., Xing, F., Wang, L., Matsumiya, T., Tanji, K., et al. (2014). ISG54 and ISG56 are induced by TLR3 signaling in U373MG human astrocytoma cells: Possible involvement in CXCL10 expression. Neuroscience Research 84, 34–42. 10.1016/j.neures.2014.03.001.

4.Warren, C.J., Griffin, L.M., Little, A.S., Huang, I.-C., Farzan, M., and Pyeon, D. (2014). The antiviral restriction factors IFITM1, 2 and 3 do not inhibit infection of human papillomavirus, cytomegalovirus and adenovirus. PLoS ONE 9, e96579. 10.1371/journal.pone.0096579.

5.Anafu, A.A., Bowen, C.H., Chin, C.R., Brass, A.L., and Holm, G.H. (2013). Interferon-inducible Transmembrane protein 3 (IFITM3) restricts reovirus cell entry. Journal of Biological Chemistry 288, 17261–17271. 10.1074/jbc.m112.438515.

6.Harper, M.S., Guo, K., Gibbert, K., Lee, E.J., Dillon, S.M., Barrett, B.S., McCarter, M.D., Hasenkrug, K.J., Dittmer, U., Wilson, C.C., et al. (2015). Interferon-A subtypes in an ex vivo model of acute HIV-1 infection: Expression, potency and effector mechanisms. PLoS Pathogens 11, e1005254. 10.1371/journal.ppat.1005254.

7. Li, K., Zhang, H., Qiu, J., Lin, Y., Liang, J., Xiao, X., Fu, L., Wang, F., Cai, J., Tan, Y., et al. (2015). Activation of cyclic adenosine monophosphate pathway increases the sensitivity of cancer cells to the oncolytic virus M1. Molecular Therapy 24, 156–165. 10.1038/mt.2015.172.

8.Khaitov, M.R., Laza-Stanca, V., Edwards, M.R., Walton, R.P., Rohde, G., Contoli, M., Papi, A., Stanciu, L.A., Kotenko, S.V., and Johnston, S.L. (2009). Respiratory virus induction of alpha-, beta- and lambda-interferons in bronchial epithelial cells and peripheral blood mononuclear cells. Allergy 64, 375–386. 10.1111/j.1398-9995.2008.01826.x.

9.Andersen, P., Pedersen, M.W., Woetmann, A., Villingshøj, M., Stockhausen, M., Ødum, N., and Poulsen, H.S. (2007). EGFR induces expression of IRF-1 via STAT1 and STAT3 activation leading to growth arrest of human cancer cells. International Journal of Cancer 122, 342–349. 10.1002/ijc.23109.

10.Reimer, T., Schweizer, M., and Jungi, T.W. (2007). Type I IFN Induction in Response to Listeria monocytogenes in Human Macrophages: Evidence for a Differential Activation of IFN Regulatory Factor 3 (IRF3). The Journal of Immunology 179, 1166–1177. 10.4049/jimmunol.179.2.1166.

11.Jacobs, J.L., Zhu, J., Sarkar, S.N., and Coyne, C.B. (2013). Regulation of mitochondrial Antiviral signaling (MAVS) expression and signaling by the mitochondria-associated endoplasmic reticulum membrane (MAM) protein GP78. Journal of Biological Chemistry 289, 1604–1616. 10.1074/jbc.m113.520254.

12.Shao, W., Earley, L.F., Chai, Z., Chen, X., Sun, J., He, T., Deng, M., Hirsch, M.L., Ting, J., Samulski, R.J., et al. (2018). Double-stranded RNA innate immune response activation from long-term adeno-associated virus vector transduction. JCI Insight 3. 10.1172/jci.insight.120474.

13.Isnardi, I., Ng, Y.-S., Srdanovic, I., Motaghedi, R., Rudchenko, S., Von Bernuth, H., Zhang, S.-Y., Puel, A., Jouanguy, E., Picard, C., et al. (2008). IRAK-4- and MYD88-Dependent pathways are essential for the removal of developing autoreactive B cells in humans. Immunity 29, 746–757. 10.1016/j.immuni.2008.09.015.

14.Yi, B., Hu, X., Zhang, H., Huang, J., Liu, J., Hu, J., Li, W., and Huang, L. (2014). Nuclear NF-κB p65 in Peripheral Blood Mononuclear Cells Correlates with Urinary MCP-1, RANTES and the Severity of Type 2 Diabetic Nephropathy. PLoS ONE 9, e99633. 10.1371/journal.pone.0099633.

15.Schöbel, A., Rösch, K., and Herker, E. (2018). Functional innate immunity restricts Hepatitis C Virus infection in induced pluripotent stem cell–derived hepatocytes. Scientific Reports 8. 10.1038/s41598-018-22243-7.

16.Perrot, I., Deauvieau, F., Massacrier, C., Hughes, N., Garrone, P., Durand, I., Demaria, O., Viaud, N., Gauthier, L., Blery, M., et al. (2010). TLR3 and Rig-Like receptor on myeloid dendritic cells and Rig-Like receptor on human NK cells are both mandatory for production of IFN-Γ in

response to Double-Stranded RNA. The Journal of Immunology 185, 2080–2088. 10.4049/jimmunol.1000532.

17.Schaefer, T.M., Desouza, K., Fahey, J.V., Beagley, K.W., and Wira, C.R. (2004). Toll-like receptor (TLR) expression and TLR-mediated cytokine/chemokine production by human uterine epithelial cells. Immunology 112, 428–436. 10.1111/j.1365-2567.2004.01898.x.

18.Kokkinopoulos, I., Jordan, W.J., and Ritter, M.A. (2004). Toll-like receptor mRNA expression patterns in human dendritic cells and monocytes. Molecular Immunology 42, 957–968. 10.1016/j.molimm.2004.09.037.

19.Hong, Y., Zhou, L., Xie, H., and Zheng, S. (2015). Innate immune evasion by hepatitis B virus-mediated downregulation of TRIF. Biochemical and Biophysical Research Communications 463, 719–725. 10.1016/j.bbrc.2015.05.130.
